# Experimental Investigations on the Structure of Yeast Mitochondrial Pyruvate Carriers

**DOI:** 10.3390/membranes12100916

**Published:** 2022-09-22

**Authors:** Ling Li, Maorong Wen, Changqing Run, Bin Wu, Bo OuYang

**Affiliations:** 1 State Key Laboratory of Molecular Biology, Center for Excellence in Molecular Cell Science, Shanghai Institute of Biochemistry and Cell Biology, Chinese Academy of Sciences, 320 Yueyang Road, Shanghai 200031, China; 2 University of Chinese Academy of Sciences, Beijing 100049, China; 3 National Facility for Protein Science in Shanghai, ZhangJiang Laboratory, Shanghai Advanced Research Institute, Chinese Academy of Sciences, Shanghai 201210, China

**Keywords:** mitochondrial pyruvate carrier (MPC), pyruvate, NMR spectroscopy, protein expression and purification, AlphaFold2

## Abstract

Mitochondrial pyruvate carrier (MPC) transports pyruvate from the cytoplasm into the mitochondrial matrix to participate in the tricarboxylic acid (TCA) cycle, which further generates the energy for the physiological activities of cells. Two interacting subunits, MPC1 and MPC2 or MPC3, form a heterodimer to conduct transport function. However, the structural basis of how the MPC complex transports pyruvate is still lacking. Here, we described the detailed expression and purification procedures to obtain large amounts of yeast MPC1 and MPC2 for structural characterization. The purified yeast MPC1 and MPC2 were reconstituted in dodecylphosphocholine (DPC) micelles and examined using nuclear magnetic resonance (NMR) spectroscopy, showing that both subunits contain three α-helical transmembrane regions with substantial differences from what was predicted by AlphaFold2. Furthermore, the new protocol producing the recombinant MPC2 using modified maltose-binding protein (MBP) with cyanogen bromide (CNBr) cleavage introduced general way to obtain small membrane proteins. These findings provide a preliminary understanding for the structure of the MPC complex and useful guidance for further studies.

## 1. Introduction

Pyruvate is an important metabolic intermediate that participates in the tricarboxylic acid (TCA) cycle to generate a large amount of energy for the physiological activities of cells [1]. Since the energy is produced in the mitochondria, pyruvate is required to be transported from the cytoplasm into the mitochondrial matrix [2]. Mitochondrial pyruvate carrier (MPC) is the key transporter located at the mitochondrial inner membrane to import pyruvate into mitochondria [3,4]. As the pyruvate gatekeeper, MPC is of fundamental importance in energy metabolism in normal cells, while the dysfunction of MPC was found to promote tumor growth in various cancer types, such as colon, brain, breast and liver cancers, and correlate with poor patient survival [5,6]. Recent studies have demonstrated that MPC inhibition also displayed the neuroprotective effects in multiple experimental models of neurodegeneration relevant to Alzheimer’s disease and Parkinson’s disease [7,8]. These discoveries have put forth MPC as a promising target for future therapeutic interventions in cancers and neurodegenerative diseases.

In most mammalian cells, MPC has been proposed to be a heterodimeric complex composed of two interacting subunits, MPC1 and MPC2 [4]. An additional subunit, MPC3 in *Saccharomyces cerevisiae* (*S. cerevisiae*) and *Arabidopsis thaliana* (*A. thaliana*), can functionally replace MPC2 [3]; no alternative MPC subunits have been reported in other higher eukaryotes. The heterodimeric composition of MPC makes it distinct from other classical mitochondrial carriers and defines a family of its own: the SLC54 family [9]. However, structure predictions previously implied that MPCs are related to the semi-SWEET family or to the SWEET family [10]. Recently, Medrano-Soto et al. proposed that the MPCs belong to the transporter-opsin-G protein-coupled receptor (TOG) superfamily [11]. Therefore, unveiling the molecular architecture of MPCs helps to gain the insights into protein assembly and function.

MPC1 and MPC2 are small integral membrane proteins, mostly containing about 100−150 amino acids. Earlier study suggested that MPC1 displays two transmembrane segments with the N- and C-termini projecting into the mitochondrial matrix, whereas MPC2 in mammals and MPC3 in yeast probably consist of three transmembrane helices, with the N-terminus projecting in the matrix and the C-terminus in the intermembrane space [12]. It was recently predicted by AlphaFold2 and RoseTTAFold that MPC1 and MPC2 both display three transmembrane helices [13]. So far, the membrane topology of MPC1 and MPC2 is still not fully resolved, as no experimental investigations on the structure of MPCs have been reported.

In this study, we aimed to determine the high-resolution structure of the MPC complex using nuclear magnetic resonance (NMR) spectroscopy, a versatile tool to solve the structures of small membrane proteins (SMPs). We first screened different constructs and successfully expressed yeast MPC1 and MPC2 in the *Escherichia coli* (*E. coli*) expression system. NMR sample conditions were established for the purified MPCs, and the assigned chemical shifts of individual yMPC1 and yMPC2 in dodecylphosphocholine (DPC) micelles showed that both subunits contain three α-helical transmembrane regions. The NMR-derived secondary structures of yMPC1 and yMPC2 differ from what were predicted by AlphaFold2. Unfortunately, a stable MPC complex was not obtained within current approaches, requiring further explorations on the high-resolution structural determination.

Interestingly, during the structural investigation of the MPC complex, we developed a new protocol for the high-level expression and purification of SMPs. Due to the low expression, high hydrophobicity and toxicity to the expression host systems, SMPs are commonly difficult targets for structural determination. Though NMR technology has shown its unprecedented capabilities in solving SMPs, it usually requires the expression of large amounts of target proteins in the *E. coli* system and purification in membrane-mimicking detergents. Previously, a TrpLE tag was introduced to express the transmembrane proteins with short sequences, typically with a length less than 80 amino acids [14]. Plenty of small transmembrane peptides in receptors or channels have been characterized using this method over the last two decades [15,16,17], and a standard protocol has been published in detail to describe step-by-step how to obtain the SMP structures at the atomic level [14]. However, for SMPs with slightly longer sequences (80–150 amino acids), the protein expression using the TrpLE fusion tag is very low, highlighting the need to develop other protocols to produce SMPs with more than 80 amino acids. Here, a modified maltose-binding protein (MBP) tag was employed together with cyanogen bromide (CNBr) cleavage to enhance the protein expression in *E. coli* and to provide high-yield protein purification with efficient tag removal.

Together, these exploratory investigations of yeast MPCs provide a preliminary structural understanding for the MPC complex. The new protocol described here could also be applicable for the recombinant expression and purification of small transmembrane proteins in general.

## 2. Materials and Methods

### 2.1. Materials

#### 2.1.1. Reagents

FastDigest restriction enzymes (BamHI, Cat. 1010BH and KpnI, Cat. 1068BH) and T4 DNA ligase for ligation (Cat. EL0016) were purchased from Thermo Fisher Scientific (Waltham, MA, USA). The Q5 polymerase for polymerase chain reaction (PCR) was from New England Biology Labs (Ipswich, MA, USA). Dodecylphosphocholine (DPC, Cat. F304) was obtained from Anatrace (Maumee, OH, USA). Ammonium chloride (^15^N, 99% (wt/wt); Cat. NLM-467), D_2_O (99.96% (vol/vol); Cat. DLM-6-PK) and D-glucose (U-^13^C6, 99% (wt/wt); Cat. CLM-1396) were obtained from Cambridge Isotope Laboratories (Tewkbury & Andover, MA, USA). Other chemicals were purchased from Amresco (Solon, OH, USA), Sigma-Aldrich (St. Louis, MO, USA) and Sangon Bioteck (Shanghai, China).

*E. coli* strains DH5α (C2987I) and BL21 (DE3) (C2527I) were purchased from New England BioLabs (Ipswich, MA, USA).

#### 2.1.2. Medium for Cell Culture

Luria–Bertani (LB) (1 L): 10 g tryptone, 5 g yeast extracts, 10 g NaCl with 100 µg/mL kanamycin sulfate.

M9 medium (1 L): 6 g Na_2_HPO_4_, 3 g KH_2_PO_4_, 1 g NH_4_Cl (^15^N, 99%, for ^15^N-labeled samples), 0.5 g NaCl, 4 g D-glucose (or 2 g 99% U-C6, for ^13^C-labeled samples), 1 mL 2 M MgSO_4_, 1 mL 100 mM CaCl_2_, 4 mL Centrum stock solution (1 tablet of Centrum dissolved in 40 mL ddH_2_O or D_2_O and then filtered). A total of 99.96% D_2_O was used for perdeuterated samples.

### 2.2. Construct Design

Sequences of *Homo sapiens (H. sapiens)* (Uniprot: Q9Y5U8 (MPC1), O95563 (MPC2)), *S. cerevisiae* (Uniprot: P53157 (MPC1), P38857 (MPC2), P53311 (MPC3)) and *Dictyostelium discoideum (D. discoideum)* (Uniprot: Q55GU4 (MPC1), Q55GU3 (MPC2)) were synthesized with codons optimized to *E. coli* codons to increase the expression (Figure S1). A His8 tag was individually fused to each protein sequence at the C-terminus. The recognition sequence Leu-Glu-Val-Leu-Phe-Gln-Gly-Pro of human rhinovirus 3C protease (HRV 3C Protease) was inserted between MPCs and His8-tag, providing a cleavage site between Gln and Gly residues. Our initial attempt to express the above constructs revealed that only MPC1 from *S. cerevisiae* (ScMPC1) with a His8 tag was highly expressed as inclusion bodies in *E. coli* BL21 (DE3). However, MPC2 and MPC3 from *S. cerevisiae* (ScMPC2, ScMPC3) with a His8-tag showed no expression. Therefore, ScMPC2 and ScMPC3 with a His8-MBP tag at the N-terminus and a 3C protease in between was further constructed, which showed very high expression in inclusion bodies analyzed by SDS-PAGE. To avoid the disulfide bond formation during the purification, all of the cysteines in ScMPC1 and ScMPC2 were mutated to serine. Further purification on ScMPC2/ScMPC3 revealed poor 3C cleavage to separate MBP and ScMPC2/ScMPC3. Therefore, cyanogen bromide (CNBr) cleavage was introduced and MBP was engineered to remove all of the methionines (MBP’). Meanwhile, S2A and M42L were introduced in ScMPC2 and M133A was introduced in ScMPC3 to avoid the side reactions on these residues during the CNBr cleavage in the purification. Mutated constructs, named yMPC1 (with C87S mutation), yMPC2 (with S2A, C85S, C111S and M42L mutations) and yMPC3 (with M133A mutation), were generated by standard PCR protocols and confirmed by DNA sequencing and used for the following studies. All primers used in this study for yMPC1, yMPC2 and yMPC3 are listed in Table S1.

### 2.3. Cloning and Protein Expression in E. coli

yMPC1-His8, His8-MBP′-yMPC2 and His8-MBP′-yMPC3 constructs were inserted into a pET-21a vector using BamHI and KpnI restriction enzyme sites and transformed into *E. coli* BL21 (DE3). The initial growth condition screening showed that yMPC1-His8, His8-MBP′-yMPC2 and His8-MBP′-yMPC3 reached the best expression with the same growth conditions described as follows. For NMR sample preparation, transformed BL21 (DE3) cells were grown overnight in 5 mL of LB media containing 100 μg/mL kanamycin sulfate at 37 °C and adapted to 50 mL M9 minimal media for 16 h. The adapted cultures were then transferred to 1 L M9 media supplemented with Centrum multivitamins and stable isotopes according to the requirement of NMR experiments, and grown at 37 °C until the optical density at 600 nm (OD_600_) reached 0.8. The cultures were induced for recombinant protein expression with 0.2 mM isopropyl β-D-thiogalatopyranoside (IPTG) at 30 °C for 16 h.

### 2.4. Purification of yMPC1

The cells were harvested and resuspended in lysis buffer (200 mM NaCl, 25 mM Tris-HCl pH 8.0), homogenized using a high-pressure homogenizer at 800 bar and centrifuged at 40,000× *g* for 30 min at 4 °C. The inclusion bodies containing yMPC1-His8 were collected and dissolved in Buffer A (200 mM NaCl, 0.5 mM β-mercaptoethanol (β-ME), 3% EMPIGEN, 25 mM Tris-HCl pH 8.0) overnight. Cell debris was removed by centrifugation at 40,000× *g* for 30 min at 4 °C. The denatured yMPC1 (from 1 L cell culture) was loaded to a 5 mL Ni-NTA column pre-equilibrated with lysis buffer and washed three times with three column volumes (CVs) of Buffer A with increasing concentrations of imidazole (10, 25, and 50 mM) and decreasing amounts of EMPIGEN (3%, 1.5%, 0%), respectively. The final washing additionally contained 2.8 mM DPC. The protein was eluted with 3 CV of elution buffer (500 mM imidazole, 2.8 mM DPC, 100 mM NaCl, 50 mM MES pH 6.5). The elution was concentrated and further purified by size exclusion chromatography (SEC) using a HiLoad 16/60 prep grade Superdex 200 GE column with SEC buffer (100 mM NaCl, 2.8 mM DPC, 0.5 mM β-ME, 50 mM MES pH 6.5). The pure protein fractions confirmed by SDS-PAGE were collected and concentrated using Centricon concentrators (EMD Millipore; MWCO, 10 kDa). The concentration of yMPC1 was determined by UV spectroscopy (280 nm; ε = 24,410 M^−1^cm^−1^). Typically, the NMR sample contains 0.5~0.8 mM yMPC1 with 100 mM NaCl, 60−100 mM DPC, 10% D_2_O and 50 mM MES pH 6.5.

### 2.5. Purification of yMPC2

The cell cultures with overexpressed His8-MBP′-yMPC2 were harvested by centrifugation at 3000× *g* for 30 min at 4 °C. The cells were lysed in the lysis buffer using the high-pressure homogenizer at 800 bar and centrifuged at 40,000× *g* for 30 min at 4 °C to collect the inclusion bodies. The protein pellets were resuspended in Buffer B (6 M guanadine-HCl (GuHCl), 100 mM NaCl, 50 mM Tris-HCl pH 8.0) at 4 °C overnight. Cell debris was removed by centrifugation at 40,000× *g* for 30 min at 4 °C. The denatured His8-MBP′-yMPC2 (from 1 L cell culture) was loaded to a 15 mL Ni-NTA column and washed three times with 3 CV of Buffer B, each time with increasing concentrations of imidazole, 10, 25, and 50 mM, respectively, and the final time additionally containing 2.8 mM DPC. The protein was eluted with 3 CV of elution buffer (6 M GuHCl, 100 mM NaCl, 500 mM imidazole, 2.8 mM DPC, 50 mM Tris-HCl pH 8.0). The elution was concentrated and further dialyzed to remove GuHCl. The precipitated His8-MBP′-yMPC2 proteins were extracted, lyophilized and then dissolved in 10 mL 80% formic acid followed by CNBr cleavage to get rid of the His8-MBP’ tag with the addition of 1 g CNBr. After CNBr removal by dialysis twice in 2 L pure water for 2 h, the protein mixture was lyophilized and dissolved in 10 mL Buffer B. The yMPC2 fragment was further separated from His8-MBP′ and uncleaved His8-MBP′-yMPC2 using nickel affinity chromatography. The flow through containing yMPC2 fragments was concentrated and refolded with the addition of 28 mM DPC and the removal of GuHCl by dialysis in dialysis buffer (100 mM NaCl, 50 mM MES pH 6.5). The dialyzed proteins were concentrated and further purified by size exclusion chromatography using the HiLoad 16/60 prep grade Superdex 200 GE column with FPLC buffer (100 mM NaCl, 2.8 mM DPC, 0.5 mM β-ME, 50 mM MES pH 6.5). The final yMPC2 NMR sample contained 0.5~0.8 mM yMPC2 (determined by UV spectroscopy (280 nm; ε = 31,970 M^−1^cm^−1^)), 100 mM NaCl, 60−100 mM DPC, 10% D_2_O, and 50 mM MES pH 6.5.

### 2.6. Purification of yMPC3

The yMPC3 protein purification followed the same protocol as yMPC2. Nickel affinity chromatography and size exclusion chromatography were performed to obtain pure yMPC3. The final yMPC3 NMR sample contained 0.2 mM yMPC3 (determined by UV spectroscopy (280 nm; ε = 31,970 M^−1^cm^−1^), 100 mM NaCl, 30−60 mM DPC, 10% D_2_O, and 50 mM MES pH 6.5.

### 2.7. SDS-PAGE

All of the proteins were examined using the Tris-Tricine SDS-PAGE system. The samples were mixed with the gel loading buffer, and stained with Coomassie Blue G250 after the electrophoresis.

### 2.8. Mass Spectrometry

Next, 0.5 μL of the purified protein solution was mixed well with 0.5 μL of matrix solution (10 mg/mL sinnapinic acid (SA), 75% acetonitrile (ACN), 25% H_2_O, 0.1% trifluoroacetic acid (TFA)). The resulting solution was spotted onto a seed layer spot on the MALDI target. The mass spectrum was collected on a 5800 MALDI-TOF/TOF (Applied Biosystems, Waltham, MA, USA).

### 2.9. NMR Spectroscopy in DPC Micelles

All NMR experiments were conducted at 37 °C on a Bruker spectrometer operating at ^1^H frequency of 600 MHz equipped with cryogenic probes. The NMR data were processed using NMRPipe [18] and analyzed using XEASY [19] and CcpNmr [20]. Sequence-specific assignment of backbone amide resonances (^1^HN, ^15^N, ^13^Cα, ^13^Cβ and ^13^C’) was accomplished using a series of gradient-selected, TROSY-enhanced triple resonance experiments, including HNCO, HN(CA)CO, HNCA, HN(CO)CA and HNCACB [21] on a uniformly [^15^N, ^13^C, ^2^H]-labeled protein. In addition, ^15^N-edited NOESY-TROSY-HSQC experiments were performed to validate the assignment. The assigned chemical shifts were used to predict the secondary structure of yMPC1 and yMPC2 using the TALOS+ program [22].

### 2.10. Circular Dichroism Spectroscopy

The purified yMPC1 and yMPC2 proteins were further characterized by circular dichroism (CD) spectroscopy using a J-715 circular dichroism spectropolarimeter. Wavelength scans were conducted from 180 to 260 nm. Experimental conditions were 16 μM of yMPC1 or 20 μM of yMPC2 in 100 mM KCl, 0.1% DPC, 50 mM K-PO4, and pH 6.5 at 298 K.

### 2.11. Pull-Down Assay

First, 450 μL 5 μM yMPC1-His8 protein solution was loaded to 100 μL nickel affinity resins. Then, yMPC2 was added to the beads in a 1:1 protein ratio and incubated on an end-over-end rotator at 4 °C for 1 h. Individual yMPC1-His8 and yMPC2 were loaded separately to the nickel beads as the controls. The beads were centrifuged for 5 min at 1500× *g*, 4 °C to remove the supernatant, and then washed using 1 mL SEC buffer three times. Finally, the bound proteins in the beads were analyzed by SDS-PAGE.

## 3. Results

### 3.1. yMPC1 Expression and Purification for NMR Spectroscopy

As mentioned in the Methods section, MPC1 from *S. cerevisiae* (ScMPC1) was selected for further studies because of its high expression in the *E. coli* system (Figure S2A). ScMPC1 consists of 130 amino acids with a calculated isoelectric point of pI 9.45. SDS-PAGE analysis showed that the His8-tag-fused ScMPC1 was expressed in inclusion bodies, which requires a critical reconstitution step to solubilize ScMPC1 into a membrane-mimetic system. Since ScMPC1 contains one cysteine (C87) in the sequence, a disulfide bond may form between two ScMPC1s during the reconstitution and disrupt the folding of ScMPC1. To avoid the disulfide bond formation, a mutation (C87S) was introduced into the protein sequence, which was named yMPC1 and used for the following structural studies.

To purify yMPC1, we followed a previously published protocol [23,24]. The first essential step in this protocol is to dissolve the target proteins in inclusion bodies with denaturing buffers containing either sarkosyl or EMPIGEN. Here, we used Dissolving Buffer A containing 200 mM NaCl, 0.5 mM β-ME, 3% EMPIGEN, 25 mM Tris-HCl pH 8.0. The solubilized yMPC1 in 3% EMPIGEN was loaded to the Ni-NTA affinity resin and subsequently refolded on column by removing the denaturant with buffer containing 2.8 mM DPC detergent. The yMPC1 eluted from the Ni-NTA resin was further concentrated and applied to SEC using a HiLoad 6/16 pg Superdex 200 column (Figure S2B). The elution profile showed a mono-dispersed and symmetric peak at an elution volume of 83.3 mL. SDS-PAGE analysis (Figure S2C) indicated that the pure fractions are homogeneous monomers and mass spectrometry (MS) (Figure S2D) showed that the molecular weight (MW) of ^15^N-yMPC1 is 17,800.38, which is consistent with the theoretical MW 17,748.12. The final yield of the recombinant yMPC1 from the *E. coli* expression system is 6 mg/L.

### 3.2. yMPC2 Expression and Purification for NMR Spectroscopy

**Figure 1:**
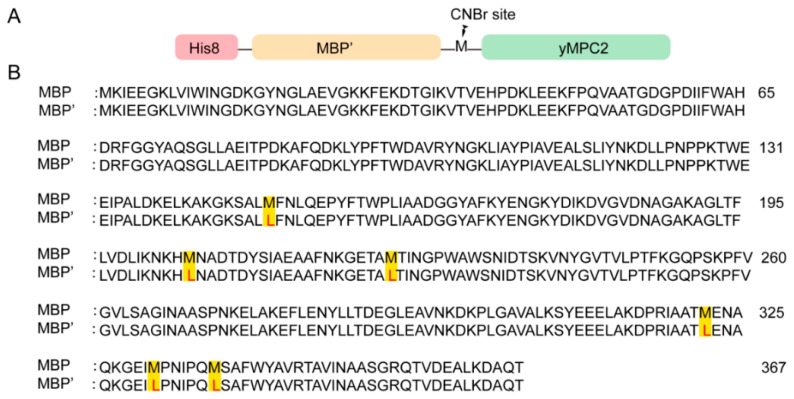
Construct of yMPC2 and the modified MBP tag. (**A**) A linear overview of the domain organization of yMPC2 construct used in this paper. (**B**) Amino acid sequence of MBP tag (residues 27–392) with the methionine residues (highlighted in yellow) mutated to leucine (red), named MBP′.

An MPC2 sequence of *S. cerevisiae* (ScMPC2) consisting of 129 amino acids was also tested with a C-terminal His8-tag initially. However, no expression was observed for ScMPC2-His8. Therefore, a MBP tag, a prokaryotic protein generally enhancing protein production and the solubility of the fusion protein [25], was inserted between an N-terminal His8-tag and ScMPC2 to increase the ScMPC2 expression. His8-MBP-ScMPC2 was highly expressed in the inclusion bodies. A 3C protease sequence was first inserted to separate MBP and ScMPC2; however, the soluble 3C protease is not stable in the strong DPC detergent, which leads to low cleavage efficiency. Therefore, CNBr cleavage with a cleavage site on a methionine residue was executed to remove the His8 and MBP tags during the purification. To avoid the CNBr cleavage on the methionine residues of MBP, all of the methionine residues in the MBP sequence (K27-T392, Uniprot: P0AEX9) were modified to leucine (MBP’) (Figure 1). Meanwhile, the methionine residue M42 in ScMPC2 was mutated to alanine. In addition, the adjacent amino acid S2 in ScMPC2 to the cleavage site M1 was mutated to alanine, since a hydroxyl or sulfhydryl group can react with the intermediate imine during the cleavage to form a homoserine and, hence, peptide bond cleavage cannot occur. To avoid the false folding caused by cysteines, all cysteines in ScMPC2 were mutated to serine, including C85S and C111S. The ScMPC2 construct with S2A, M42L, C85S and C111S mutations was named yMPC2 and used for the next structural exploration.

**Figure 2:**
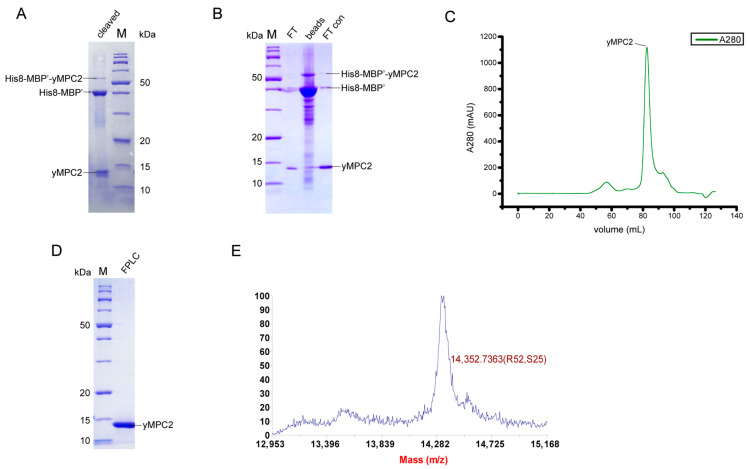
Protein purification of yMPC2. (**A**) SDS-PAGE analysis of the CNBr cleavage products of His8-MBP′-yMPC2. The protein bands were detected by Coomassie blue staining and are indicated by a line. (**B**) SDS-PAGE analysis of the protein purity after removing uncleaved His8-MBP′-yMPC2 and His8-MBP′-tag by nickel affinity resins. The labels in the figure are indicated as below: “FT” is the flow through; “beads” is the supernatant from the boiled nickel affinity resin; and “FT con” is the concentrated flow through. (**C**) Size exclusion chromatography of yMPC2 in SEC buffer (100 mM NaCl, 2.8 mM DPC, 0.5 mM β-ME, 50 mM MES pH 6.5) using a HiLoad 16/60 pg Superdex 200 GE column. (**D**) SDS-PAGE analysis of the elution peak from size exclusion chromatography in (**C**). (**E**) Mass spectrometry analysis of the purified ^14^N-yMPC2 protein. The experimental molecular mass is 14,352.74 Da, consistent with the theoretical molecular mass of 14,357.49 Da.

The yMPC2 was purified using a totally different protocol from yMPC1 (Figure S3). The His8-MBP′-yMPC2 inclusion bodies were denatured in Dissolving Buffer B (100 mM NaCl, 0.5 mM β-ME, 6 M GuHCl, 50 mM Tris-HCl pH 8.0) and loaded onto a Ni-NTA column, and then eluted by imidazole. The eluted His8-MBP′-yMPC2 was dialyzed to remove the denaturant GuHCl, and then protein precipitants were dissolved in 80% formic acid (FA) and cleaved by CNBr (Figure 2A). After removing FA and CNBr by dialysis, the protein mixtures were lyophilized and dissolved again in Dissolving Buffer B. A second Ni affinity chromatography was performed to remove the His8-MBP′ tag and the uncleaved fusion proteins (Figure 2B). The flow through, mainly containing yMPC2, was collected and reconstituted by adding a sufficient amount of DPC and dialyzing in Buffer D (100 mM NaCl, 50 mM MES pH 6.5). The yMPC2 protein was further purified by SEC using a HiLoad 6/16 pg Superdex 200 column, showing a single sharp peak at 82.5 mL (Figure 2C). The pure fractions from SEC were collected and verified by SDS-PAGE and mass spectrometry (Figure 2D,E), showing that ^14^N-yMPC2 is a homogeneous monomer with MW of 14,352.74, which is consistent with the theoretical MW 14,357.49. A typical yield for yMPC2 production is ~3 mg/L.

### 3.3. yMPC3 Expression and Purification for NMR Spectroscopy

The yMPC3 was purified using the same protocol as for yMPC2 (Figure S4) with a yield of 3.5 mg/L. However, the SEC file showed an asymmetric elution peak (Figure S4A), indicating that the yMPC3 protein was not homogeneous. The SDS-PAGE analysis showed that the fractions of yMPC3 contained different kinds of degraded proteins (Figure S4B).

### 3.4. Secondary Structure Determination of MPC1 and MPC2

**Figure 3:**
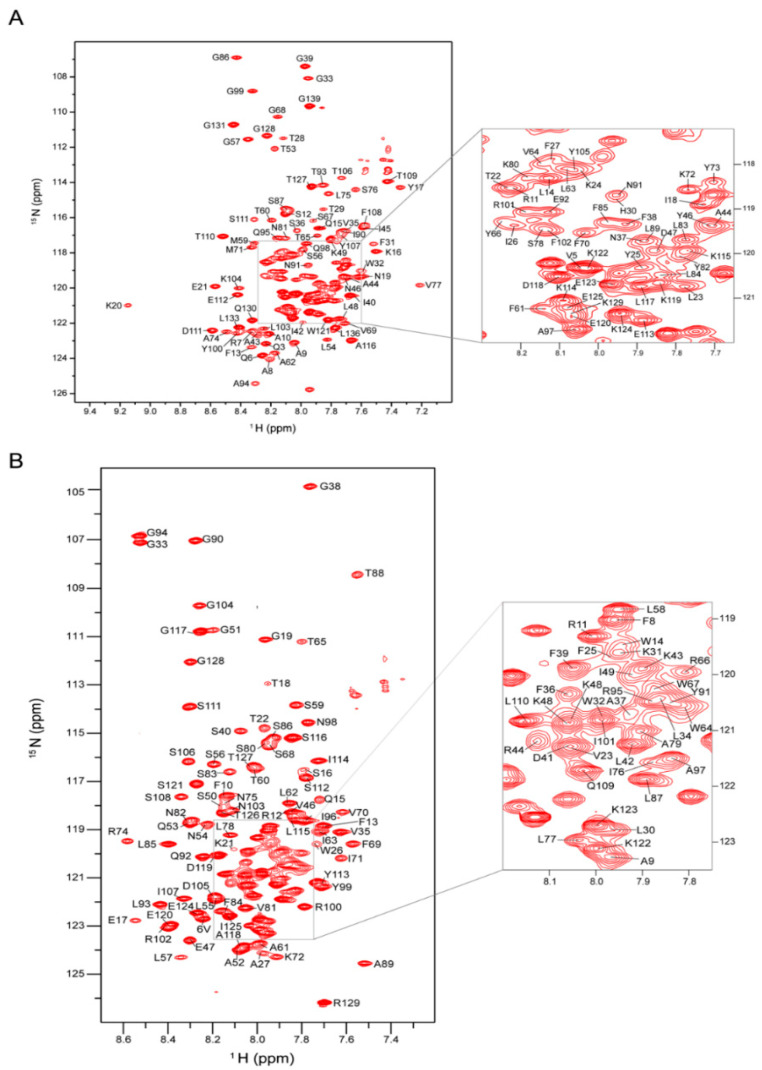
NMR spectra of yMPC1 and yMPC2 with backbone assignments. (**A**) 2D ^1^H-^15^N TROSY-HSQC spectrum of yMPC1 in DPC micelles with backbone resonances assigned. The spectrum was recorded at ^1^H frequency of 600 MHz using [^15^N, ^13^C, ^2^H]-labeled protein. The crowded region is magnified on the right. (**B**) Two-dimensional ^1^H-^15^N TROSY-HSQC spectrum of yMPC2 in DPC micelles with backbone resonances assigned. The spectrum was recorded at ^1^H frequency of 600 MHz using [^15^N, ^13^C, ^2^H]-labeled protein. The crowded region is magnified on the right.

The purified proteins in the DPC micelles were further concentrated to make the final NMR samples containing 0.5~0.8 mM yMPC1 or yMPC2 in NMR buffer (100 mM NaCl, 60−100 mM DPC, 10% D_2_O, 50 mM MES pH 6.5). The NMR spectroscopy was performed on a Bruker spectrometer at ^1^H frequency of 600 MHz equipped with cryogenic probes and 37 °C. The two-dimensional (2D) ^1^H-^15^N transverse relaxation optimized spectroscopy–heteronuclear single-quantum coherence (TROSY-HSQC) correlation spectrum of yMPC1 showed good resolution and dispersion and yMPC2 gave a more congregate spectrum (Figure 3A,B). As expected from the results of the SEC and SDS-PAGE, yMPC3 showed a much worse spectrum with aggregated peaks and plenty of resonances missing (Figure S4C). Therefore, further NMR characterizations were only performed on yMPC1 and yMPC2.

**Figure 4:**
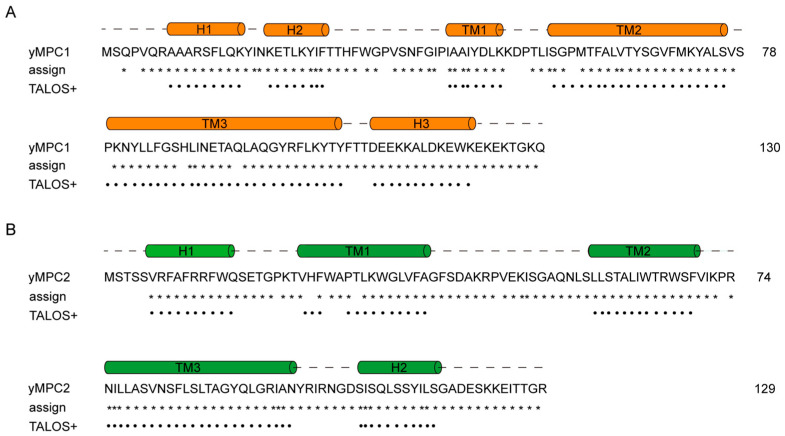
Secondary structures of yMPC1 and yMPC2 from TALOS + program. (**A**) Secondary structure of yMPC1 predicted based on TALOS+ analysis. The assigned residues are indicated as asterisks, and the helical regions predicted by the assigned chemical shift are indicated by black dots. (**B**) Secondary structure of yMPC2 predicted based on TALOS+ analysis. The assigned residues are indicated as asterisks, and the helical regions predicted by the assigned chemical shift are indicated by black dots.

The backbone assignments of yMPC1 and yMPC2 were determined by triple resonance NMR experiments using uniformly [^13^C, ^15^N, 80% ^2^H]-labeled proteins. The assigned backbone resonances of ^1^H, ^13^C and ^15^N nuclei were further confirmed by ^15^N-edited nuclear Overhauser effect spectroscopy (NOESY). For yMPC1, we assigned 94% of ^15^N and ^1^HN resonances, as well as 94% of ^13^Cα and ^13^Cβ signals (Figure 3A). A secondary structure prediction from the TALOS+ program [22] using the assigned chemical shifts indicated α-helical content in the transmembrane (TM) regions including residues I42-K49, S56-S76 and P79-Y107; the soluble domains contain three short helices with residues A8-K16, K20-F27 and D111-K122, and the rest of the protein is random coils (Figure 4A). For yMPC2, we assigned 95% of the backbone resonances of non-proline residues (Figure 3B). Secondary structure prediction analysis was also performed using the TALOS+ program and indicated a relatively dynamic TM1 (V23-A37) with weak signals, TM2 (L57-F69) and TM3 (N75-N98) (Figure 4B). Two short helices were identified in the soluble part consisting of residues V6-Q15 and S106-S116, respectively.

We next mixed yMPC1 and yMPC2 to form the heterodimer. However, the SEC profile showed no shift on the elution volume of the mixed proteins, indicating no larger complex had formed. The pull-down assay using nickel affinity resins also showed that yMPC1-His8 and yMPC2 were not detected together in the eluted fractions, which confirmed that no strong interaction exists between yMPC1 and yMPC2.

### 3.5. CD Characterization of yMPC1 and yMPC2

The yMPC1 and yMPC2 proteins in the NMR samples were further characterized by CD spectroscopy. As shown in Figure S5, both proteins generated good CD spectra with typical α-helical characteristics. The spectrum has two negative bands at 222 and 208 nm, and a positive band at ~190 nm for an all-helix protein, indicating that the proteins were well folded.

### 3.6. Secondary Structure Comparison of yMPC1 and yMPC2 from NMR and AlphaFold2

We compared the secondary structures of the yMPC1 and yMPC2 determined from our NMR data with the secondary structures predicted by AlphaFold2, a powerful computational method on protein structure prediction published recently. Both results showed that yMPC1 contains three transmembrane helices, two amphipathic helices and a C-terminal water-soluble helix. The major differences between the experimental results and the representative structure model of yMPC1 from AlphaFold2 come from the first TM helix; NMR data showed that T29–P41 are very dynamic with no rigid helical arrangement, while AlphaFold2 predicted a helix through T29–K49 (Figure 4A and Figure S6A). On the other hand, yMPC2 displayed more disagreement on the secondary structures between NMR and AlphaFold2. yMPC2 also gave a shorter helical arrangement on TM1 in the NMR results. G51-S56 and Y99-N103 are not folded into helix. Moreover, the last C-terminal helix including residues E120–T127 is non-helical in the NMR calculation (Figure S6B).

## 4. Discussion

The MPC complex has an important role in energy production, but very few investigations on its structure have been performed. In this study, we expressed and purified yMPC1, yMPC2 and yMPC3 using two different purification protocols. A typical protocol for the expression and purification of mitochondrial carriers, such as uncoupling protein 1 (UCP1) [23], the short Ca^2+^-binding mitochondrial carrier (SCaMC) [24], ADP/ATP carrier (AAC) [26] and uncoupling protein 2 (UCP2) [27], was applied to yMPC1. In contrast, yMPC2 and yMPC3 were expressed and purified in a unique way with a His8-MBP′ tag expression, but CNBr cleavage to remove the MBP’ tag, where the MBP’ tag is an engineered MBP tag for which all methionine residues are removed to accommodate with CNBr cleavage. Meanwhile, the CNBr cleavage can tolerate the harsh denaturing conditions and achieve high cleavage efficiency. To this end, high yields of yMPC2 and yMPC3 were obtained in minimal media with isotopic labeling for NMR experiments. We believe that our protocol using the modified MBP tag combined with CNBr cleavage will be useful as an alternative method to generate milligram quantities of small membrane proteins.

We further completed the backbone chemical shift assignments for yMPC1 and yMPC2, resulting in the secondary structures calculated by the TALOS+ program. The TM1 helix in both yMPC1 and yMPC2 showed intrinsic flexibility and dynamics in the individual monomers, indicating that TM1 in both protein subunits may be involved in the interface of the complex or play an important role in the pyruvate transport. The secondary structures from NMR results are different from those of the AlphaFold2 prediction. AlphaFold2 has shown its ability to accurately predict three-dimensional (3D) models of soluble protein structures. However, because of the lack of sufficient membrane protein structures, the prediction accuracy on membrane proteins is low. Recent literature using RoseTTAFold to calculate the 3D structure of MPCs also showed a different secondary structure from the NMR results [13]. Though both programs gave the prediction of the tertiary structure of the MPC complex, the differences of the secondary structures between the predictions and experimental results implicate that further structural characterization of the MPC complex is needed.

Unfortunately, we failed to obtain the yMPC complex when yMPC1 and yMPC2 are both reconstituted in DPC micelles. DPC is one of the most common detergents used in solution NMR to solubilize membrane proteins. However, the use of DPC is still in debate, since DPC is a relatively strong detergent. Our previous studies showed that DPC-reconstituted UCP1 [23] and SCaMC [24] preserved their activities, indicating that DPC detergent is a viable membrane-mimetic system to study mitochondrial carriers. However, the choice of DPC micelles to characterize the MPC complex here is not a workable case, possibly due to DPC’s strong charge properties, or because MPC1 and MPC2 indeed have weak interactions in the heterodimeric conformation. In addition, we cannot exclude the possibility that the mutations we introduced in the constructs may disrupt the interaction interface, since cysteine residues were shown to be essential in mitochondrial carrier dimerization and transport activity [28,29]. Though further experiments are needed to identify suitable sample conditions for the MPC complex, our study here provides the details of purifying yMPC1 and yMPC2, and the first-hand structural investigations on MPC, which offers valuable information for MPC structure determination.

## Data Availability

Not applicable.

## Supplementary Materials

The following supporting information can be downloaded at: https://www.mdpi.com/article/10.3390/membranes12100916/s1, Figure S1: Sequence alignment of MPC from different species; Figure S2: Protein expression and purification of yMPC1; Figure S3: Purification flowchart of yMPC2 and yMPC3; Figure S4: Purification and NMR spectrum of yMPC3; Figure S5: CD spectroscopy of yMPC1 (A) and yMPC2 (B); Figure S6: Comparison of the secondary structures of yMPC1 (A) and yMPC2 (B) from NMR data and predicted by AlphaFold2; Table S1: Primers used to produce the constructs of yMPC1, yMPC2 and yMPC3 in this study.
