# Supplementary material for: Experimental Investigations on the Structure of Yeast Mitochondrial Pyruvate Carriers
Source: Membranes (Basel). 2022 Sep 22;12(10):916. doi: 10.3390/membranes12100916 (PMC9608981; doi:10.3390/membranes12100916)
Supplement: Supplementary file 1 [file membranes-12-00916-s001.zip › membranes-1911026-supplementary.pdf]

membranes

MDPI

Article

Experimental Investigations on the Structure of Yeast Mitochondrial Pyruvate Carriers

Ling Li [1,2], Maorong Wen [1], Changqing Run [1], Bin Wu [3] and Bo OuYang [1,2,*]

1 State Key Laboratory of Molecular Biology, Center for Excellence in Molecular Cell Science, Shanghai Institute of Biochemistry and Cell Biology, Chinese Academy of Sciences, 320 Yueyang Road, Shanghai 200031, China; liling2017@sibcb.ac.cn (L.L.); mrwen@sibcb.ac.cn (M.W.); runchangqing@sibcb.ac.cn (C.R.)
2 University of Chinese Academy of Sciences, Beijing 100049, China
3 National Facility for Protein Science in Shanghai, ZhangJiang Laboratory, Shanghai Advanced Research Institute, Chinese Academy of Sciences, Shanghai 201210, China; bin.wu@sibcb.ac.cn
* Correspondence: ouyang@sibcb.ac.cn

Citation: Li, L.; Wen, M.; Run, C.; Wu, B.; OuYang, B. Experimental Investigations on the Structure of Yeast Mitochondrial Pyruvate Carriers. *Membranes* **2022**, *12*, 916. https://doi.org/10.3390/membranes12100916

Academic Editors: Ayman El-Hattab and Mohammed Almannai

Received: 27 August 2022
Accepted: 14 September 2022
Published: 22 September 2022

Publisher's Note: MDPI stays neutral with regard to jurisdictional claims in published maps and institutional affiliations.

Copyright: © 2022 by the authors. Licensee MDPI, Basel, Switzerland. This article is an open access article distributed under the terms and conditions of the Creative Commons Attribution (CC BY) license (https://creativecommons.org/licenses/by/4.0/).

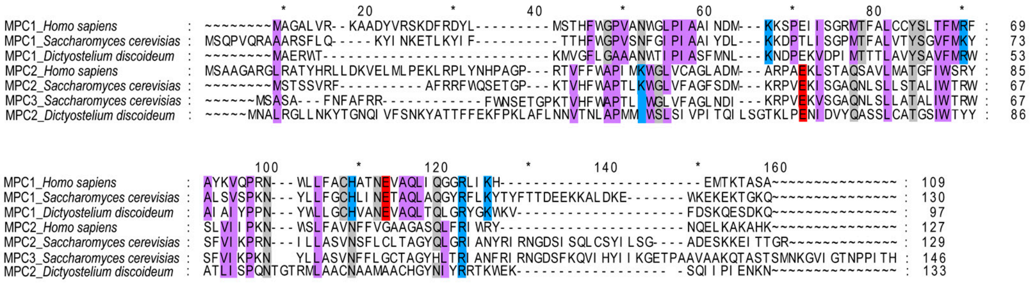

Figure S1. Sequence alignment of MPC from different species. Sequence comparison between MPC1 and MPC2, including *Homo sapiens* (Uniprot: Q9Y5U8 (MPC1), O95563 (MPC2)), *Saccharomyces cerevisiae* (Uniprot: P53157 (MPC1), P38857 (MPC2), P53311 (MPC3)) and *Dictyostelium discoideum* (Uniprot: Q55GU4 (MPC1), Q55GU3 (MPC2)). The conserved amino acids are highlighted.

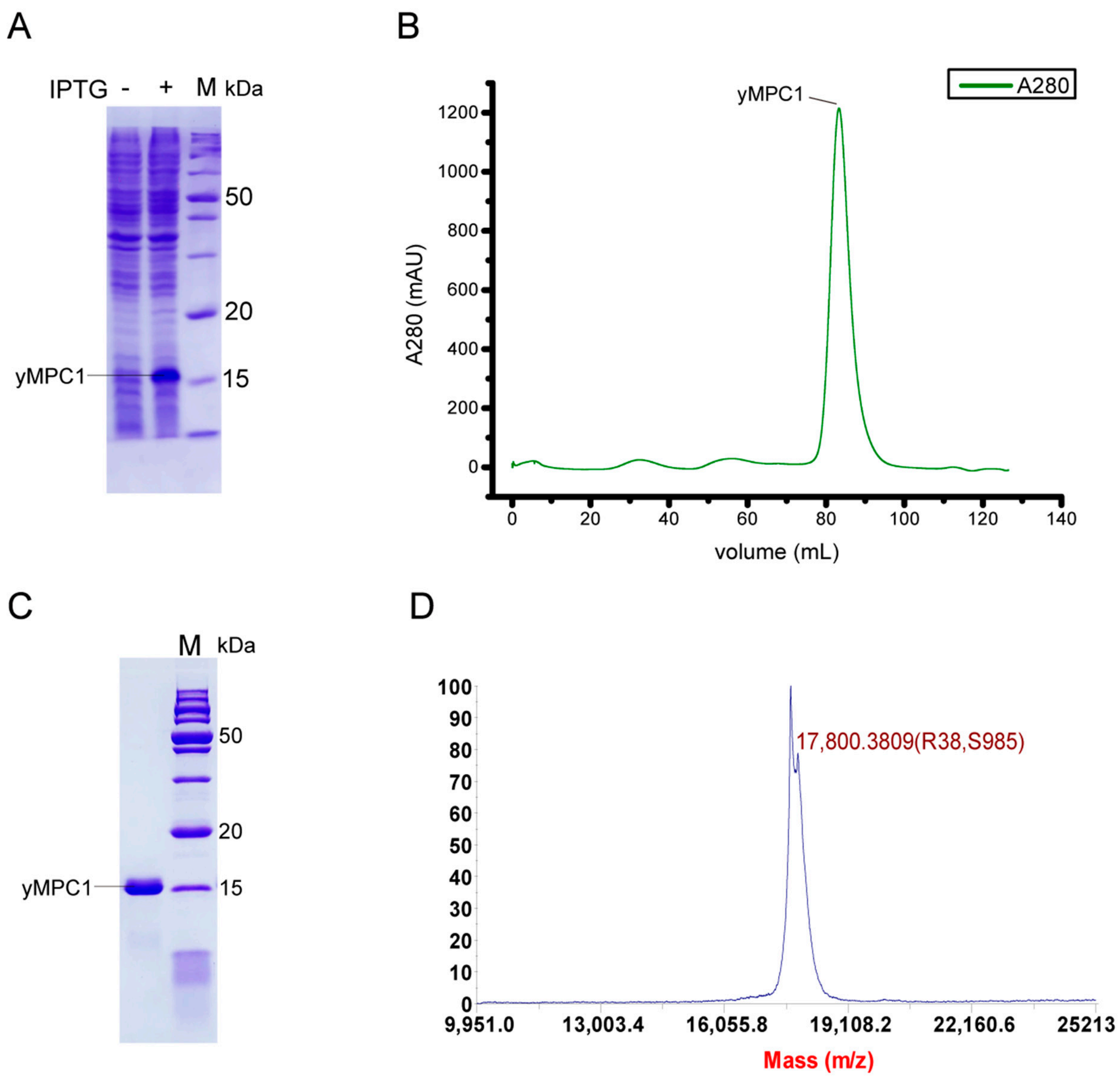

Figure S2. Protein expression and purification of yMPC1. (A) SDS-PAGE analysis of yMPC1 expression, cells without (lane -) or with (lane +) IPTG induction were examined. After IPTG induction, yMPC1 showed high expression. (B) Size exclusion chromatography of yMPC1 in SEC buffer (100 mM NaCl, 2.8 mM DPC, 0.5 mM β-ME, 50 mM MES pH 6.5) using a HiLoad 16/60 prep grade Superdex 200 GE column. (C) SDS-PAGE analysis of the elution peak from the size exclusion chromatography in (B) showing the purity of yMPC1. (D) Mass spectrometry analysis of the purified ^{15}N-yMPC1 protein. The experimental molecular mass is 17,800.38 Da, consistent with the theoretical molecular mass of 17,748.12 Da.

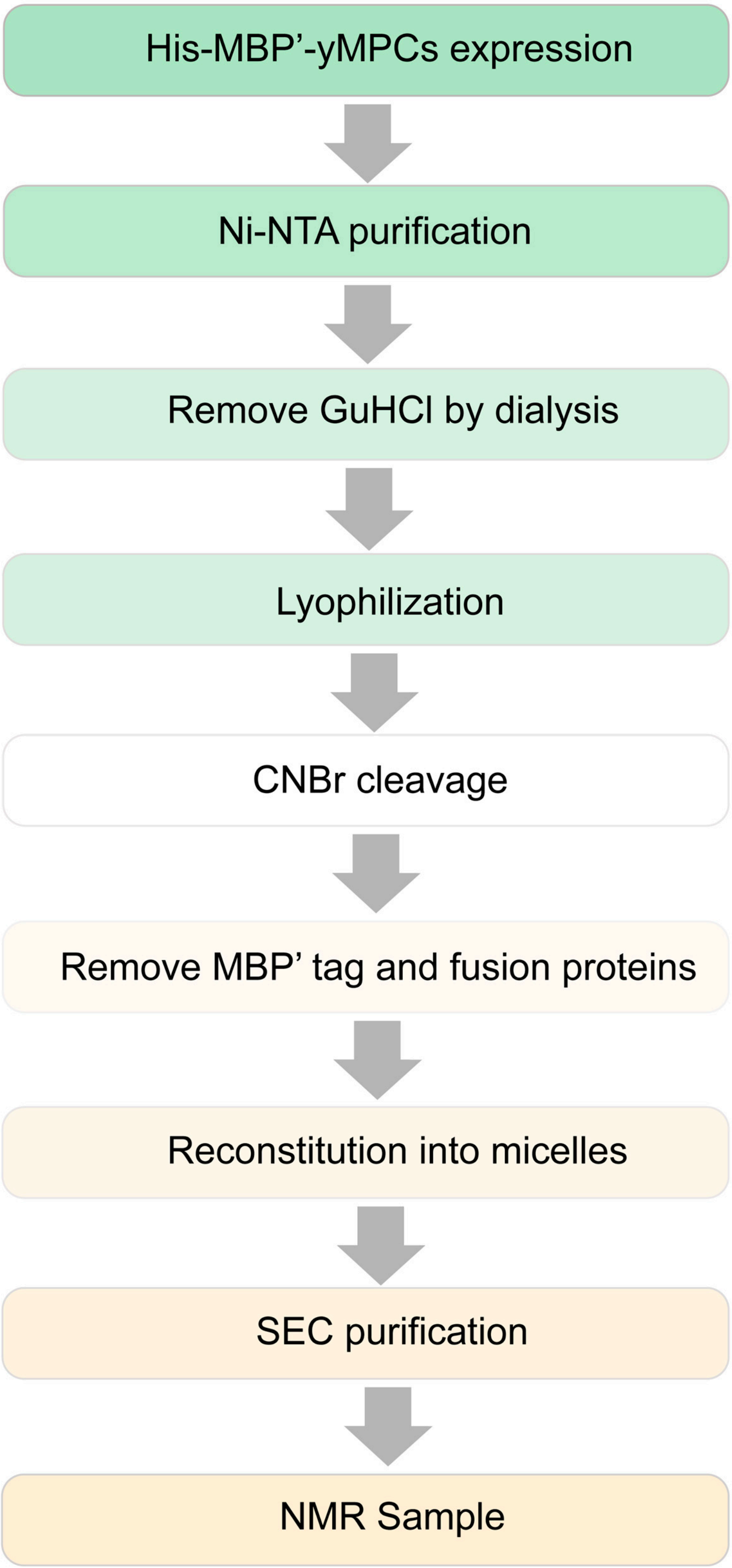

Figure S3. Purification flowchart of yMPC2 and yMPC3. The steps required for yMPC2 and yMPC3 expression, purification and reconstitution into micelles to obtain a final NMR sample.

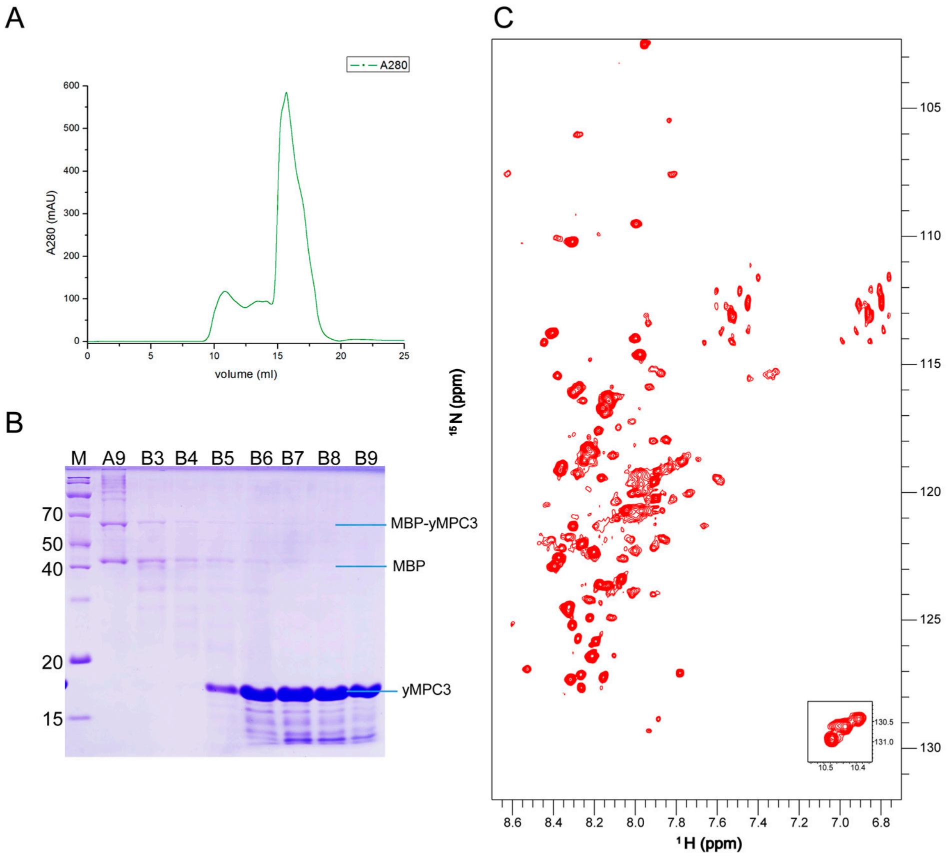

Figure S4. Purification and NMR spectrum of yMPC3. (A) Size exclusion chromatography of yMPC3 in SEC buffer (100 mM NaCl, 2.8 mM DPC, 0.5 mM β-ME, 50 mM MES pH 6.5) using a Superdex 200 10/300 GL GE column. (B) SDS-PAGE analysis of yMPC3 fractions from the SEC purification. (C) 2D ^{1}H-^{15}N TROSY-HSQC spectrum of yMPC3 in DPC micelles. The spectrum was recorded at ^{1}H frequency of 700 MHz using ^{15}N-labeled protein. The sidechain signals were put on the right bottom.

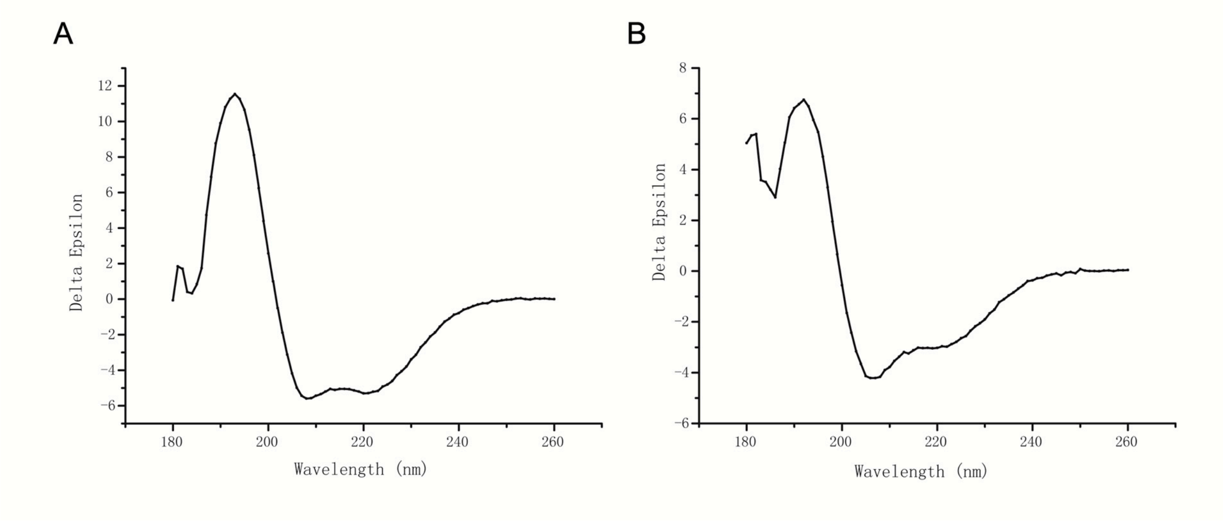

Figure S5. CD spectroscopy of yMPC1 (A) and yMPC2 (B). Samples for CD spectra were prepared similarly as NMR samples but changed into CD buffer（100 mM KCl, 50 mM K-PO_4, pH 6.5, 0.1% DPC) by PD-10 to remove Na^+. CD signals were recorded in a 0.1 mm path length cuvette at room temperature on Chirascan V100 (Applied Photophysics). Signals were recorded from 260 nm to 180 nm with 1 nm step size and processed using a Chirascan analysis software.

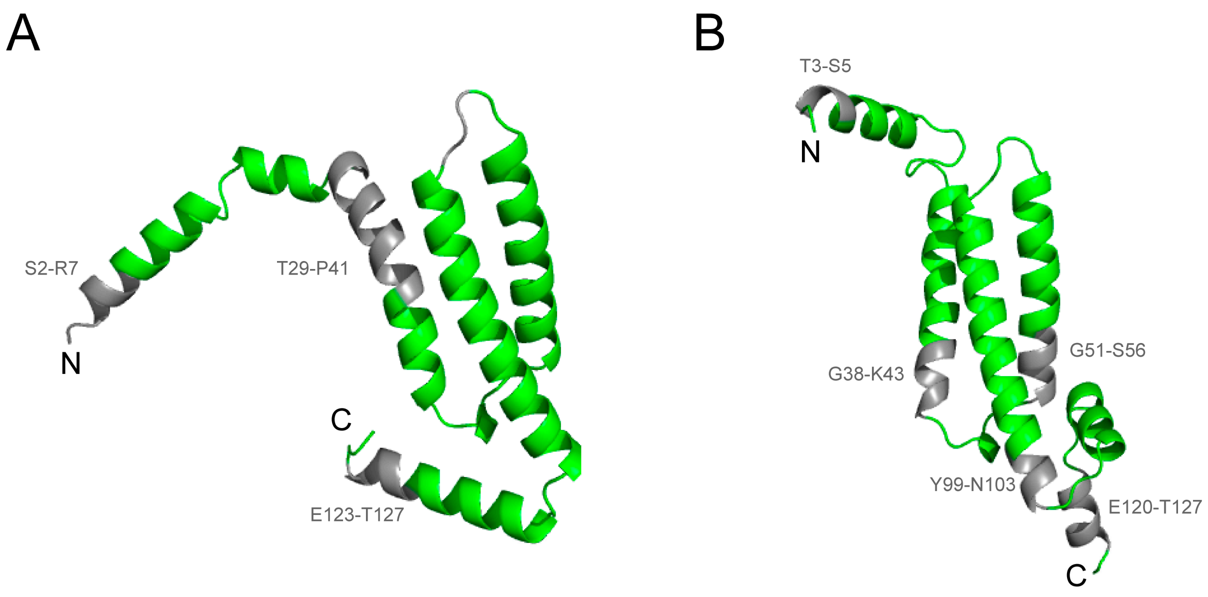

Figure S6. Comparison of the secondary structures of yMPC1(A) and yMPC2 (B) from NMR data and predicted by AlphaFold2. The secondary structures with the consistency are colored in green, the regions with the differences are colored in grey.

Table S1. Primers used to produce the constructs of yMPC1, yMPC2 and yMPC3 in this study.

yMPC1 Primers	Primer sequences
His-yMPC1F	CGGGATCCATGAGTCAGCCGGTTCA
His-yMPC1R	GGGGTACCCTGTTTGCCGGTCTTTTCTT
C87S-F	CGAAAAATTATCTGCTGTTTGGTAGTCATCTGATTAATGAAAC-CGC
C87S-R	GCGGTTTCATTAATCAGATGACTACCAAACAGCAGA-TAATTTTTCG
yMPC2 Primers	Primer sequences
His-MBP-yMPC2F	CGGGATCCATGAGTACCAGTAGCGTT
His-MBP-yMPC2R	GGGGTACCTCAGCGACCGGTGGTAATTTC
S2A-F	GGCCCGGGATCCATGGCTACCAGTAGCGTTCG
S2A-R	CGAACGCTACTGGTAGCCATGGATCCCGGGCC
M42L-F	GACGGATCCATGAGTACCAGTAGC
M42L-R	GACGGTACCTCAGCGACCGG
C85S-F	GCGTGAATAGTTTTCTGAGCCTGACCGCAGGTTATC
C85S-R	GATAACCTGCGGTCAGGCTCAGAAAACTATTCACGC
C111S-F	GATAGCATTAGTCAGCTGAGCAGTTATATTCTGAGC
C111S-R	GCTCAGAATATAACTGCTCAGCTGACTAATGCTATC
yMPC3 Primers	Primer sequences
His-MBPF	GGTACCTGAGATCCGGCTGCTAACAAAG
His-MBPR	GGATCCCGGGCCCTGAAACAGCACTT
MBP-yMPC3F	TGCTGTTTCAGGGCCCGG-GATCCATGAGCGCCAGCGCGTTCAACTT
MBP-yMPC3R	TTAGCAGCCGGATCTCAGGTACCTTAGTGGGTAATCGGCG-GATTGGTGC
M133A-F	CAAACAAACCGCGAGCAC-CAGCGCGAACAAAGGCGTGATCGGCAC
M133A-R	GTGCCGATCACGCCTTTGTTCGCGCTGGTGCTCGCGGTTT-GTTTG
